# Relationship between extraversion and mental health literacy in Chinese adolescents: a chain mediation model

**DOI:** 10.3389/fpsyg.2025.1562788

**Published:** 2026-01-08

**Authors:** Zhanfang Liu, Fangru Yuan, Jianzheng Du

**Affiliations:** 1 Xiangnan Preschool Education College, Chenzhou, China; 2 Linyi Middle School of Chenzhou, Chenzhou, China; 3 School of Education, Guangzhou University, Guangzhou, China

**Keywords:** adolescents, chain mediation, extraversion, mental health literacy, psychological help-seeking, social support

## Abstract

**Background and aim:**

Approximately one-quarter of the global population experiences mental disorders, with onset frequently occurring during adolescence. Low mental health literacy (MHL) is a crucial barrier to help-seeking. Although extraversion, social support, and psychological help-seeking all facilitate access to mental health information, their combined and sequential roles in influencing MHL remain underexplored. Using the Capability, Opportunity, Motivation, and Behavior (COM-B) model, this study examined the serial mediating roles of social support and psychological help-seeking in the relationship between extraversion and MHL in Chinese adolescents.

**Methods:**

A cross-sectional online survey was conducted involving 482 adolescents (ages 16–19; 56.43% girls) from Hunan Province, China. The participants completed the Extraversion Personality Questionnaire, Social Support Rating Scale (SSRS), General Help-Seeking Questionnaire (GHSQ), and Mental Health Literacy Questionnaire (MHLQ). Data were analyzed using correlation and chain mediation analyses (bootstrapping with 5,000 samples), while controlling for gender and birthplace.

**Results:**

(1) Extraversion, social support, psychological help-seeking, and MHL were all positively correlated with each other (*p* < 0.001). (2) Path analysis revealed that social support (β = 0.072, 95% CI [0.033, 0.125]) and psychological help-seeking (β = 0.037, 95% CI [0.013, 0.072]) independently mediated the relationship between extraversion and MHL. (3) Additionally, these factors also serially mediated this relationship (β = 0.046, 95% CI [0.026, 0.073]). However, the direct effect of extraversion on MHL was not significant (β = 0.021, 95% CI [−0.071, 0.113]), indicating full mediation.

**Conclusion:**

Social support and psychological help-seeking sequentially and fully mediate the relationship between extraversion and MHL in adolescents. Enhancing social support systems and promoting proactive help-seeking may effectively improve MHL, even among individuals with low extraversion.

## 1 Introduction

Approximately one-quarter of the world’s population experiences some form of mental illness. In approximately 75% of cases, the first onset occurs between the ages of 14 and 25 (Casañas et al., 2022). Despite the serious impacts of mental illness on patients’ health, interpersonal relationships, and academic well-being (Baxter et al., 2022), only a minority of affected individuals seek professional help, and delaying treatment imposes an even greater burden and cost (Kim et al., 2020; Osman et al., 2022). Several studies have suggested that the primary barriers to help-seeking are a lack of knowledge about mental illness, stigmatizing attitudes, and other related factors, all of which reflect low mental health literacy (MHL) (Campos et al., 2022; Casañas et al., 2022). MHL encompasses an individual’s knowledge and beliefs regarding the prevention, recognition, and management of mental illnesses (Jorm et al., 1997). In China, MHL, as promoted by the China National Healthcare Commission, is considered one of the most fundamental, cost-effective, and efficient measures for improving the mental health of the entire population (Ming and Chen, 2020).

While promoting MHL, especially among youth, is a clear priority, understanding the factors that contribute to its development remains an active area of inquiry. Previous studies have identified several potential influencing factors, such as extraversion, social support, and help-seeking attitudes (Ding and Li, 2021; Xie et al., 2021; Wang et al., 2023). However, studies to date have typically examined these variables only in isolation or in pairwise relationships, leaving a gap in our understanding of their potential combined effects on MHL. Specifically, the potential chain mechanisms through which a personality trait such as extraversion might translate into higher MHL—for example, through the potential mediating effects of enhanced social support and a greater willingness to seek help—have not been comprehensively tested. Addressing this gap is crucial for developing more nuanced and effective interventions. Therefore, this study aims to investigate the complex relationships between extraversion, social support, psychological help-seeking, and MHL in adolescents by proposing and testing a sequential mediation model to elucidate the underlying pathways.

## 2 Literature review and hypothesis development

### 2.1 Extraversion and MHL

Extraversion can positively influence an individual’s MHL through multiple pathways. According to social cognitive theory, extraverts are skilled in social interaction (Yan and Yao, 2025); therefore, they have more opportunities to engage with others and discuss mental health topics. An empirical study by Ding and Li supports this view, showing that extraverted individuals tend to actively seek mental health information through various channels, particularly interpersonal ones such as reaching out to friends and teachers, thereby acquiring rich knowledge of mental health in social settings (Ding and Li, 2021).

### 2.2 Social support as a mediator

Social support refers to the material and emotional support and assistance that individuals receive from external sources such as family, friends, colleagues, or the community (Feng et al., 2024). Wang et al. found that social support positively predicts MHL, suggesting that strong social support offers effective interpersonal resources; the more support an individual receives, the more psychological resources they can obtain from their social network (Wang et al., 2023). Furthermore, Zhang’s research elaborates on this mechanism by demonstrating that social support can stimulate demand for both relational and growth-oriented mental health services, thereby promoting the development of MHL (Zhang, 2024). Individuals with extraverted personalities exhibit traits such as sociability, activeness, optimism, and enthusiasm, all of which are associated with strong social skills (Zou and Zhang, 2006). These skills, in turn, facilitate the establishment and maintenance of social relationships, creating social networks through which these individuals can gain support and assistance (Fan and Liu, 2019). Therefore, it is plausible that extraversion leads to greater perceived social support, which in turn fosters enhanced MHL.

### 2.3 Psychological help-seeking as a mediator

Psychological help-seeking is the process through which individuals seek help from others to address their psychological problems, primarily from mental health professionals, such as psychologists and psychiatrists, but can also include non-professionals such as parents, teachers, classmates, and friends (Shui et al., 2021). MHL encompasses the knowledge, skills, and attitudes related to mental health that are acquired through developmental experience and environmental interactions (Chen and Li, 2024). According to social-ecological systems theory, individual development arises from ongoing interactions with one’s environment (Yao and Cao, 2023), and psychological help-seeking represents a key form of such active engagement (Liu and Li, 2021). Moreover, several studies have found that individuals who have sought professional psychological help tend to exhibit higher levels of MHL (Jiang et al., 2021; Huang and Li, 2022). Therefore, through help-seeking, individuals can not only obtain relevant information from their environment but also adjust their cognitive patterns and establish positive health beliefs and behaviors, thereby fostering the development and enhancement of their MHL.

### 2.4 The serial mediation pathway

As outlined above, the strong social skills associated with extraversion enable individuals to obtain greater social support (Bao et al., 2018), which in turn provides more opportunities to access psychological and material resources from their social networks. Since social support positively predicts the intention to seek psychological help (Li and Wang, 2024), and willingness to seek help allows individuals to acquire mental health knowledge and skills through social relationships (Xie et al., 2021), this process ultimately contributes to the development of their MHL. While the relationships among these factors have been explored individually, their integration into a coherent mediating chain remains underinvestigated.

### 2.5 Hypothesized theoretical model and hypotheses

Based on the above synthesis, it is hypothesized that social support and psychological help-seeking act as serial mediators in the relationship between extraversion and MHL. The specific hypotheses are as follows:

H1: Extraversion is positively associated with MHL.

H2: Social support mediates the relationship between extraversion and MHL.

H3: Psychological help-seeking mediates the relationship between extraversion and MHL.

H4: Social support and psychological help-seeking are chain mediators in the relationship between extraversion and MHL (i.e., Extraversion → Social Support → Help-Seeking Willingness → MHL).

**Figure 1:**
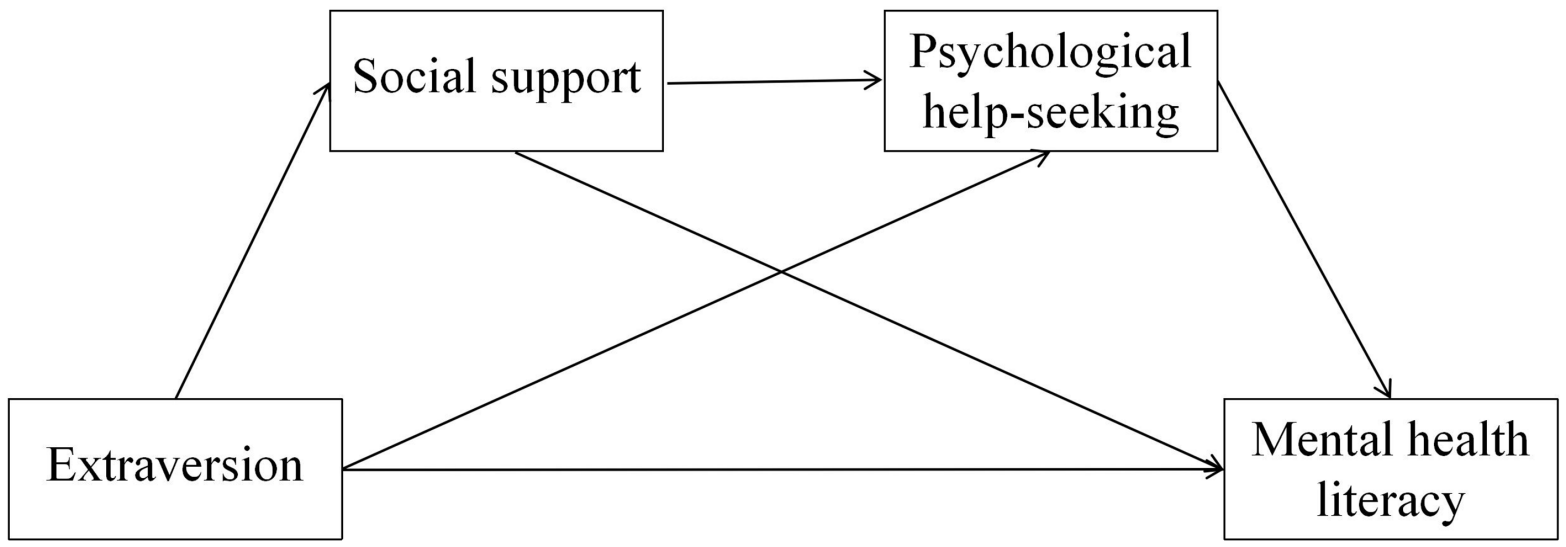
Hypothesized chain mediation model linking extraversion to mental health literacy via social support and psychological help-seeking.

A hypothesized model corresponding to H4 is presented in Figure 1.

## 3 Materials and methods

### 3.1 Participants

Convenience sampling was used to select 504 adolescents from a school in Hunan Province, who then completed a series of online questionnaires, including the Mental Health Literacy Questionnaire (MHLQ). After the questionnaires were collected, 22 invalid questionnaires were excluded based on the following criteria: excessively short completion times (i.e., less than the cutoff value of 250 s, determined from a pre-test in which the fastest completion time among seven participants was 250 s) and inappropriately regular responses (i.e., selecting the same option more than 15 times consecutively) (Wu, 2020). The remaining 482 valid questionnaires were retained, yielding a validity rate of 95.63%. Among the valid respondents, 210 (43.57%) were boys and 272 (56.43%) were girls. Their ages ranged between 16 and 19 years old, with a mean age of (17.43 ± 0.58); and 387 (80.29%) were from rural areas and 95 (19.71%) were from urban areas.

### 3.2 Instruments

#### 3.2.1 Extraversion

Extraversion was measured using the extraverted personality questionnaire developed by Wang et al. (2011). The questionnaire comprises eight items scored on a six-point Likert scale ranging from 1 (*very non-conforming)* to 6 (*very conforming*), with two items reverse-scored. A sample item is, “I enjoy attending social and entertainment gatherings.” The Cronbach’s α for this questionnaire was 0.82 in the current study.

#### 3.2.2 Social support

The Chinese version of the Social Support Rating Scale (SSRS), as revised by Xiao (1994), was used. This scale comprises 10 items and assesses three dimensions: subjective support (4 items), objective support (3 items), and support utilization (3 items). The total score was used to evaluate social support, with higher values indicating greater perceived social support. A sample item from the objective support dimension is, “How many close friends do you have from whom you can seek support and help?” (Response options: 0, 1–2, 3–5, and 6 or more). The Cronbach’s for the SSRS in this study was 0.70.

#### 3.2.3 Psychological help-seeking

The General Help-Seeking Questionnaire (GHSQ) was used to assess the likelihood that respondents would seek psychological help from their parents, teachers, friends (including classmates or romantic partners), or mental health professionals (e.g., psychologists, counsellors). All items were scored on a seven-point Likert scale ranging from 1 (*extremely unlikely*) to 7 (*extremely likely*) (Wilson et al., 2005). A sample item is, “If you were experiencing psychological distress that you could not resolve yourself, how likely would you be to seek help from your parents?” The Cronbach’s α for the GHSQ was 0.73 in this study.

#### 3.2.4 MHL

The Mental Health Literacy Questionnaire (MHLQ) developed by Wu (2020) assesses six factors: knowledge of mental health, knowledge of mental illness, attitudes and habits related to maintaining and promoting one’s own mental health, attitudes and habits for coping with one’s own mental illness, attitudes and habits for maintaining and promoting others’ mental health, and attitudes and habits for coping with others’ mental illness. The MHLQ consists of 60 items. Items 1 to 30 (e.g., “Emotional stability is a sign of mental health,”) are scored as either 1 or 0 (*yes* = 1, *no* = 0, *do not know* = 0), while items 31–60 (e.g., “I think my mental health is the most important,”) are scored on a five-point Likert scale, and these scores are then converted to 0 and 1, with ratings of 4 or above re-coded as 1 for positively worded items, when calculating the total score. The maximum total score is 60, with higher scores indicating better MHL. The Cronbach’s α for the MHLQ was 0.81 in this study.

#### 3.2.5 Covariates

In the current study, gender (1 = *boy*, 2 = *girl*) and birthplace (1 = *rural*, 2 = *urban*) were included as covariates, as prior studies have found that gender and birthplace are statistically significantly associated with MHL (Ming et al., 2019).

### 3.3 Statistical analyses

Data were processed and analyzed using Jamovi 2.3.21 (The Jamovi Project, 2022). Statistical analyses included a common method bias test, descriptive statistics, correlation matrix analysis, and mediation analysis. Specifically, a chain mediation analysis was conducted to examine the serial mediating roles of social support and general psychological help-seeking in the relationship between extraversion and MHL while controlling for gender and birthplace. Bootstrapping of regression estimates with 5,000 samples and a 95% confidence interval was conducted. An effect was considered significant if the 95% confidence interval did not include zero.

## 4 Results

### 4.1 Common method bias test

Several procedural measures were implemented in this study to mitigate the possibility of common method bias, including anonymous administration of the questionnaire, reverse-scoring of selected items, and the use of varied response formats (i.e., true/false, Likert). Harman’s single-factor test was used to assess common method bias. The results of exploratory factor analysis indicated that 14 factors had eigenvalues greater than 1. The first factor accounted for 6.68% of the total variance, which was below the cutoff value of 40%. Therefore, common method bias was not a serious issue for this study (Tang and Wen, 2020).

### 4.2 Preliminary and correlation analyses

Table 1 presents the results of the correlation analysis of extraversion, social support, psychological help-seeking, and MHL. All four of these variables were significantly and positively correlated with each other (*p* < 0.001).

**Table 1:** Correlations among extraversion, social support, psychological help-seeking, and mental health literacy.

Variables	M	SD	1	2	3	4
1. Extraversion	28.58	6.91	—			
2. Social Support	26.65	4.56	0.41^***^	—		
3. Psychological Help-Seeking	18.47	4.55	0.34^***^	0.53^***^	—	
4. Mental Health Literacy	37.17	7.31	0.18^***^	0.32^***^	0.34^***^	—

^*^p < 0.05, ^**^p < 0.01, ^***^p < 0.001.

### 4.3 Tests of mediation effects

**Figure 2:**
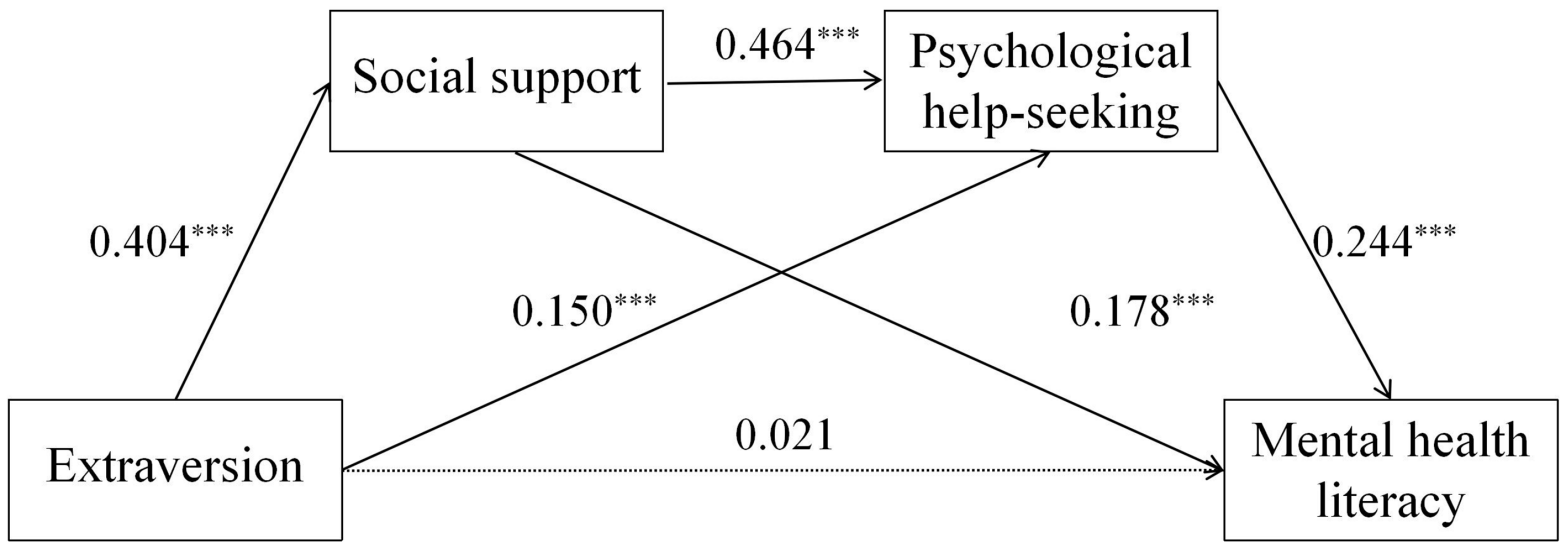
The chain mediation model of social support and psychological help-seeking between extraversion and mental health literacy (MHL).

The mediating effects of social support and psychological help-seeking on the relationship between extraversion and MHL were examined using the Jamovi 2.3.21 software, controlling for gender and birthplace in a generalized linear model. The results (see Table 2; Figure 2) are as follows:

**Table 2:** Results of the serial mediation analysis.

Type	Effect	Estimate	SE	95% C.I		β	z	*p*
				Lower	Upper			
Indirect	EX → SS → MHL	0.076	0.023	0.033	0.125	0.072	3.244	0.001
	EX → PH → MHL	0.039	0.015	0.013	0.072	0.037	2.519	0.012
	Gender1 → SS → MHL	0.046	0.117	−0.182	0.294	0.003	0.396	0.692
	Gender1 → PH → MHL	−0.008	0.141	−0.303	0.267	−0.001	−0.057	0.955
	BP1 → SS → MHL	−0.025	0.147	−0.319	0.273	−0.001	−0.17	0.865
	BP1 → PH → MHL	−0.242	0.176	−0.611	0.081	−0.013	−1.375	0.169
	EX → SS → PH → MHL	0.049	0.012	0.026	0.073	0.046	4.025	< 0.001
	Gender1 → SS → PH → MHL	0.029	0.073	−0.115	0.178	0.002	0.405	0.686
	BP1 → SS → PH → MHL	−0.016	0.093	−0.205	0.16	−0.001	−0.172	0.863
Component	EX → SS	0.267	0.029	0.21	0.323	0.404	9.059	< 0.001
	SS → MHL	0.285	0.081	0.129	0.438	0.178	3.527	< 0.001
	EX → PH	0.099	0.031	0.04	0.159	0.15	3.188	0.001
	PH → MHL	0.392	0.084	0.227	0.559	0.244	4.667	< 0.001
	Gender1 → SS	0.162	0.392	−0.593	0.937	0.018	0.413	0.679
	Gender1 → PH	−0.02	0.353	−0.737	0.659	−0.002	−0.058	0.954
	BP1 → SS	−0.088	0.503	−1.057	0.88	−0.008	−0.175	0.861
	BP1 → PH	−0.618	0.426	−1.462	0.202	−0.054	−1.451	0.147
	SS → PH	0.464	0.043	0.38	0.545	0.464	10.86	< 0.001
Direct	EX → MHL	0.022	0.046	−0.071	0.113	0.021	0.467	0.641
	Gender1 → MHL	1.023	0.638	−0.243	2.286	0.069	1.603	0.109
	BP1 → MHL	0.57	0.756	−0.896	2.051	0.031	0.754	0.451
Total	EX → MHL	0.185	0.047	0.092	0.278	0.175	3.902	< 0.001
	Gender1 → MHL	1.091	0.659	−0.201	2.383	0.074	1.654	0.098
	BP1 → MHL	0.287	0.822	−1.324	1.897	0.016	0.349	0.727

EX, Extraversion; PH, Psychological Help-Seeking; SS, Social Support; MHL, Mental Health Literacy. Gender was coded as 1 = boy, 2 = girl; Birthplace (BP) was coded as 1 = rural, 2 = urban. Bootstrap confidence intervals (percentile method, 5,000 resamples) are reported. β represents the fully standardized effect size.

First, the constructed chain mediation model is presented in Figure 2, with detailed path coefficients and their significance levels provided in Table 2. Specifically, extraversion positively predicted both social support (β = 0.404, *p* < 0.001) and psychological help-seeking (β = 0.150, *p* = 0.001). Social support, in turn, not only positively predicted MHL (β = 0.178, *p* < 0.001) but also positively predicted psychological help-seeking (β = 0.464, *p* < 0.001). Furthermore, psychological help-seeking positively predicted MHL (β = 0.244, *p* < 0.001). The predictive effects of the control variables (gender and birthplace) were not significant for any of the tested paths (all *p* > 0.05).

Second, the bootstrap analysis (with 5,000 resamples) revealed that the independent mediating effect of social support was significant (β = 0.072, 95% CI [0.033, 0.125]); the independent mediating effect of psychological help-seeking was also significant (β = 0.037, 95% CI [0.013, 0.072]); and the chain mediating effect of these two variables was likewise significant (β = 0.046, 95% CI [0.026, 0.073]). However, the direct effect of extraversion on MHL was not significant (β = 0.021, 95% CI [−0.071, 0.113]). This indicated that the sequential mediation chain of social support and psychological help-seeking fully mediated the relationship between extraversion and MHL.

## 5 Discussion

This study reveals the intrinsic chain mediation mechanism linking extraversion to MHL. The results show that extraversion does not exert a direct effect on MHL, but rather an indirect effect through the sequential mediation of social support and the willingness to seek psychological help.

### 5.1 The non-significant direct effect of extraversion on MHL

The direct path linking extraversion to MHL was not significant; this result may stem from their differing natures. The core of MHL is knowledge and skills (Amer and Hamdan-Mansour, 2025), whereas extraversion is a social tendency and emotional style (Chen, 2010). According to social cognitive theory, while extraverts do not inherently possess greater mental health knowledge, their broader social networks lead to more frequent exposure to relevant information and discussions, thereby providing more learning opportunities. In other words, the advantage of extraversion lies in creating “social channels” for acquiring knowledge, and the internalization of knowledge must be achieved through the “transportation” of knowledge via these channels. Social support and the willingness to seek psychological help are the key carriers of this “transportation” process. Therefore, the link between extraversion and MHL is likely to be essentially social and procedural, rather than cognitive and direct.

### 5.2 The chain mediating effects of social support and general psychological help-seeking

The findings of this study indicate that social support and psychological help-seeking sequentially mediate the relationship between extraversion and MHL. The Capability, Opportunity, Motivation, and Behavior (COM-B) model posits that behaviors arise from dynamic interactions among ability, opportunity, and motivation (Michie et al., 2011). Ability encompasses the physical and mental skills and knowledge (such as social skills) that an individual needs to have to perform a specific behavior; opportunity refers to external factors that facilitate or hinder a behavior (such as social support); and motivation is the psychological force that drives the behavior (such as interest or need) (Li et al., 2025). Therefore, to systematically explain the above-mentioned mediating paths, this study introduced the COM-B model and integrated its empirical findings into the theoretical framework of “capability-opportunity-motivation” to achieve an in-depth interpretation of the internal mechanism. The starting point of this theoretical chain lies in the strong social skills (capability) that extraverted individuals possess, which enable them to effectively establish and maintain interpersonal relationships, thereby securing more abundant social support (opportunity) for themselves (Liu and Huang, 2010). Social support not only provides emotional companionship but also serves as an important source of mental health information, thereby further enhancing their “opportunities.” Moreover, by reducing stigma and creating a demonstration effect, social support enhances individuals’ motivation to actively seek professional help when experiencing psychological distress (Jansem et al., 2025). Ultimately, this strong motivation to seek help drives individuals to take specific actions, such as consultation or information search, enabling them to systematically enhance their MHL through professional feedback and active learning. This analysis, based on the COM-B model, further explains the possible bidirectional relationship between the willingness to seek help and MHL (Xu et al., 2021; Yang et al., 2024).

### 5.3 Research contributions and implications

This study makes two key theoretical contributions. First, it confirms the independent mediating roles of social support and the willingness to seek psychological help in the relationship between extraversion and MHL. Second, and more importantly, it provides a systematic theoretical explanation for this link, using the COM-B model, by revealing the chain mediating mechanism involving these two variables. This finding suggests that, at a practical level, even for introverted individuals, consciously building a social support system and normalizing the act of seeking psychological assistance can effectively promote the development of their MHL.

### 5.4 Limitations

This study has some shortcomings worth noting. First, the use of convenience sampling from a single school in Hunan Province, along with data collected at a single time point, limits the representativeness of the sample and the generalizability of the findings. Second, self-report measures are susceptible to social desirability bias, which may have led the participants’ responses to deviate from their true opinions (Wu et al., 2024). Third, the cross-sectional design does not permit causal inferences to be drawn regarding the relationships between the variables. Fourth, the analysis controlled for only a limited set of covariates (gender and birthplace) and did not account for other potential confounding factors, such as family socioeconomic status. Finally, regarding statistical methods, the analysis conducted using the Jamovi software did not include tests of structural validity (e.g., confirmatory factor analysis), and common method bias was assessed solely using Harman’s single-factor test, which may be insufficient. Future research could address these limitations by employing more diverse sampling, incorporating longitudinal or experimental designs, controlling for a broader range of theory-relevant covariates, and utilizing additional statistical methods.

## 6 Conclusion

In conclusion, this study observed significant positive correlations among extraversion, social support, psychological help-seeking, and MHL. Moreover, social support and psychological help-seeking sequentially mediated the relationship between extraversion and MHL. These findings suggest that interventions aimed at enhancing social support systems and promoting proactive help-seeking may effectively improve MHL in adolescents, even among those with low levels of extraversion.

## Data Availability

The raw data supporting the conclusions of this article will be made available by the authors, without undue reservation.
